# Molecule doping enables high-quality perovskite junctions for all-perovskite tandem solar cells

**DOI:** 10.1093/nsr/nwae170

**Published:** 2024-05-16

**Authors:** Lingeswaran Arunagiri, Feng Gao

**Affiliations:** Department of Physics, Chemistry and Biology (IFM), Linköping University, Sweden; Department of Physics, Chemistry and Biology (IFM), Linköping University, Sweden

The maximum theoretical power conversion efficiency (PCE) for single-junction solar cells based on the Shockley-Queisser (S-Q) model is 33.7% for an optimal bandgap (Eg) of 1.34 eV under 1-sun illumination [1]. Multi-junction (tandem) solar cells present a realistic option for overcoming the S-Q limit and achieving efficiency of >40% due to decreased thermalization loss in tandem configuration [2]. Metal halide perovskites, in particular, are the most sought-after materials for tandem solar cells (TSCs), as their bandgap energy can be fine-tuned from 1.2 to 3 eV by simple compositional engineering. Recently, two-junction perovskite tandems with silicon attained an astounding efficiency of 33.9%, surpassing the previous record of PCEs of single-junction perovskite and silicon solar cells, and bolstering investor confidence in perovskite-based tandem technologies [3].

All-perovskite (perovskite/perovskite) TSCs, made by stacking a narrow-bandgap (typically 1.2 eV) perovskite subcell on top of a wide-bandgap (typically 1.8 eV) perovskite subcell, are gaining popularity in this context. They are particularly attractive owing to their simple fabrication procedures, which include low-temperature solution processing, and their ability to employ lightweight flexible substrates that can help in lowering manufacturing costs [4]. However, the PCE of all-perovskite TSCs lags behind perovskite/silicon tandems, due to significant voltage and fill factor (FF) losses in the wide-bandgap perovskite subcell, resulting in mediocre performance [5]. As a result, increasing open-circuit voltage (V_OC_) and FF in wide-bandgap perovskite solar cells (PSCs) is of great technological interest. One straightforward approach is to construct an intermixed 2D/3D heterojunction with a post-treatment on the 3D perovskite film surface to increase doping density [6]. However, so far, only a moderate doping density of 10^13^ cm^−3^ has been obtained, which is insufficient to generate a high interfacial electric field and improve carrier extraction [7].

**Figure 1.**
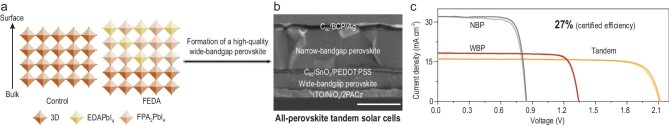
(a) Schematic of perovskite structures for control and FEDA-treated surfaces. The FEDA-treated surface is created with a 0.5 : 0.5 weight ratio of 3-fluorophenethylammonium iodide (FPA) and ethane-1,2-diammonium iodide (EDA). (b and c) Device structure of all-perovskite TSCs and their corresponding *J-V* curve.

Recently, a new study led by Zhijun Ning and Qingqing Ji addresses this issue by creating a high-quality perovskite junction via a post-surface treatment approach that increases the carrier density to 10^16^ cm^−3^ [8]. Their approach includes the formation of n-type low-dimensional perovskite on top of wide-bandgap 3D perovskite (1.77 eV) utilizing FEDA (Fig. 1a), a diammonium and monoammonium molecule combination. The incorporation of FEDA effectively manipulates the doping profile of the perovskite surface, resulting in a high FF of >83% and a V_OC_ of 1.34 V due to a decreased interfacial barrier, which improves charge extraction while minimizing interface charge recombination. Following this result, as seen in Fig. 1b and c, the authors employed FEDA-treated wide-bandgap perovskite to produce all-perovskite TSCs in combination with a ∼1.25 eV narrow-bandgap perovskite subcell, obtaining a certified efficiency of over 27%, ranking among the best for all-perovskite tandems. Furthermore, the authors demonstrated increased operational stability for all-perovskite TSCs as a result of better perovskite heterojunction quality, which eliminated undesired charge accumulation.

Overall, this timely study addresses one of the most challenging aspects of all-perovskite TSCs in the formulation of highly efficient and stable wide-bandgap perovskites, paving the way for an experimentally feasible technique to break the 30% efficiency barrier and, perhaps, win the ‘race’ for low-cost tandem technology.
